# Indoor radon exposure in Africa: A critical review on the current research stage and knowledge gaps

**DOI:** 10.3934/publichealth.2025020

**Published:** 2025-03-13

**Authors:** Leonel J.R. Nunes, António Curado

**Affiliations:** proMetheus, Unidade de Investigação em Materiais, Energia e Ambiente para a Sustentabilidade, Instituto Politécnico de Viana do Castelo, Rua da Escola Industrial e Comercial de Nun'Alvares, 4900–347 Viana do Castelo, Portugal

**Keywords:** indoor radon exposure, public health, health risks, African countries, radon research

## Abstract

Indoor radon exposure poses significant health risks and has prompted testing and mitigation programs in regions such as Europe, North America, Japan, and South Korea. However, African countries have not adopted similar measures on a comparable scale. Limited research on radon exposure in Africa can be attributed to a lack of awareness among policymakers and the public, insufficient expertise in radiation protection and measurements, and restricted access to resources such as laboratories and testing equipment. This review examines existing research articles on radon exposure conducted in African countries, focusing on the efforts made by specific nations, such as Tunisia and Sudan, to address this issue. It analyzes the scope, scale, and impact of these initiatives compared to global efforts in managing radon exposure risks. The findings reveal that the study of radon exposure in Africa is still in its early stages, with limited progress and modest initiatives compared to other regions. While some efforts have been made, they are insufficient to effectively address the significant health risks associated with radon exposure. There is an urgent need for African policymakers and researchers to prioritize radon exposure as a public health issue. Developing frameworks, standards, and mitigation strategies is essential to reduce risks in homes and workplaces. This review emphasizes the importance of addressing radon exposure in African countries and offers recommendations to guide future research and policy development.

## 1. Introduction

In recent years, there has been growing concern worldwide about the health risks associated with exposure to radon gas, especially indoor radon [1]. Radon, a naturally occurring radioactive gas, has been classified by the World Health Organization (WHO) and the International Agency for Research on Cancer (IARC) as a Group 1 carcinogen. It is the second leading cause of lung cancer after cigarette smoke, particularly in poorly ventilated environments. The European Commission issued the 2013/59/Euratom Directive, setting a reference level of 300 Bq/m³ for indoor radon concentration, which has been adopted by several countries. This directive mandates the measurement of radon levels in both private homes and workplaces.

Many countries in Europe, North America, Japan, and South Korea have taken steps to assess and reduce the risks associated with radon exposure [2]. Indoor radon is of a particular concern because it is a leading cause of lung cancer, ranking second after tobacco smoking [3]. When radon gas is released from the ground, it can seep into buildings and accumulate at high concentrations, putting occupants at risk [4]. In response to this, many countries have introduced radon testing and mitigation programs, aimed at reducing the concentration of radon in homes and other indoor environments [5]. In Europe, the European Union (EU) has set a reference level of 300 Bq/m³ for the indoor radon concentration [6]. Member states are encouraged to take measures to reduce radon levels in homes and other buildings, including testing, remediation, and public awareness campaigns [7]. Many countries, such as Finland, Sweden, and Switzerland, have already implemented national radon action plans, which include measures to reduce radon exposure in homes and workplaces [8]. Similarly, in North America, the United States Environmental Protection Agency (EPA) recommends that homeowners test for radon and take action if levels are found to be high, generally defined as concentrations exceeding 200–300 Bq/m³, as recommended by organizations such as the World Health Organization (WHO) and the EU. These thresholds are based on studies that linked prolonged exposure to elevated radon levels with an increased risk of lung cancer [9]. The EPA has set an action level of 148 Bq/m³, above which remediation is recommended [10]. In Canada, radon testing is recommended in all homes, and the government has set a guideline level of 200 Bq/m³ for indoor radon [11]. In Japan and South Korea, where radon exposure is also a concern, the governments have introduced regulations to protect citizens from exposure to high levels of radon [2].

Along with the growing concern regarding the health risks posed by radon exposure, there has been a surge in scientific research on the assessment, monitoring, mitigation, and remediation of radon in Europe, North America, Japan, and South Korea [12]. In recent years, numerous studies have been conducted to determine the extent of indoor radon levels, identify potential sources of radon, and develop effective mitigation strategies [13]. The EU has taken a leading role in regulating radon exposure and promoting research on the subject [12]. The EU adopted Directive 2013/59/Euratom in 2013, which established a set of standards for radon levels in buildings and required member states to implement measures to reduce radon exposure [7]. This has led to a significant increase in radon research in Europe, with numerous studies being conducted on radon levels in homes, schools, and workplaces [14]. In North America, both the United States and Canada have established guidelines for radon exposure and require radon testing in certain circumstances [15]. This has led to an increase in radon research in these countries, with a focus on identifying radon sources and developing effective mitigation strategies. The United States Environmental Protection Agency (EPA) has been particularly active in promoting radon research and awareness, with a variety of resources available for homeowners, researchers, and policymakers [16].

Additionally, Japan and South Korea have taken steps to address radon exposure, with both countries implementing regulations for radon levels in buildings [17]. In Japan, the Ministry of Health, Labour and Welfare established guidelines for radon levels in 2000, and ongoing research continues to investigate potential sources of indoor radon and effective mitigation strategies [18]. In South Korea, the Ministry of Environment has established guidelines for radon levels in buildings, and research has focused on identifying sources of radon in homes and developing effective mitigation strategies [19]. In all of these regions, the concern over radon exposure has led to an increase in scientific research on the topic. Researchers are investigating the health risks associated with radon exposure, thereby identifying potential sources of radon and developing effective mitigation strategies.

Despite the growing concern regarding the risks associated with radon exposure in some parts of the world, there are still regions where little attention has been given to the study and monitoring of radon. One such region is the African continent, where only a small number of countries have devoted any effort toward the assessment and regulation to address indoor radon risk exposure. The reasons for this lack of attention in African countries are complex and multifaceted. First, radon is still a relatively underexplored topic in Africa, and researchers have mainly focused on other environmental pollutants. There is a lack of awareness among policymakers and the general public regarding the health risks associated with radon exposure, and as a result, little attention has been given to its study. Furthermore, the lack of studies on radon in these countries may be due to the lack of experts and trained personnel in the field of radiation protection and measurement. Moreover, there may be a shortage of facilities and equipment required to measure the radon levels in these countries. Another factor that contributes to the limited research on radon in African countries is the limited resources available to these countries. Many of these countries face significant economic challenges, and their resources are often directed toward more pressing concerns, such as poverty reduction, infrastructure development, and infectious disease control. Funding for research in radiation protection and measurement may not be a priority, and as a result, radon research may be considered a low priority. Despite these challenges, some African countries have made efforts to address the issue of indoor radon exposure. For example, Tunisia has conducted studies on radon levels in phosphate factories and their environments, as well as in some dwellings and schools [20]. Moreover, Sudan has conducted studies on indoor radon levels in homes and workplaces, as well as the soil gas radon concentrations in some locations [21]. However, the number of studies and the scale of the effort is still limited compared to other regions of the world.

The objective of this review is to examine research articles conducted on the African continent, focusing on the primary subjects addressed in these studies. A thorough search was conducted on the Scopus database, which is a reputable database for scientific literature, to determine the extent of research efforts and the current state of knowledge regarding indoor radon exposure in various African countries. This analysis not only seeks to identify the key areas of concern in the existing literature but also strives to uncover potential knowledge gaps and research opportunities that could be explored in future studies. By delving into the current body of research, this review aims to provide a holistic understanding of indoor radon exposure on the African continent, thus shedding light on the underlying factors contributing to this issue and the potential health implications for the affected populations. Ultimately, the insights gleaned from this review may contribute to the development of more effective strategies, policies, and interventions to mitigate the risks associated with indoor radon exposure in Africa.

## 2. Materials and methods

To conduct this review, a systematic literature search was conducted using the Scopus database with the following query: (“radon” AND “indoor”) AND “name of each African country”. This search yielded articles published between 1990 and 2023, which focused on indoor radon exposure across the African continent. Duplicate records were removed, and articles were screened based on the relevance to radon exposure in residential or occupational settings.

**Figure 1.**
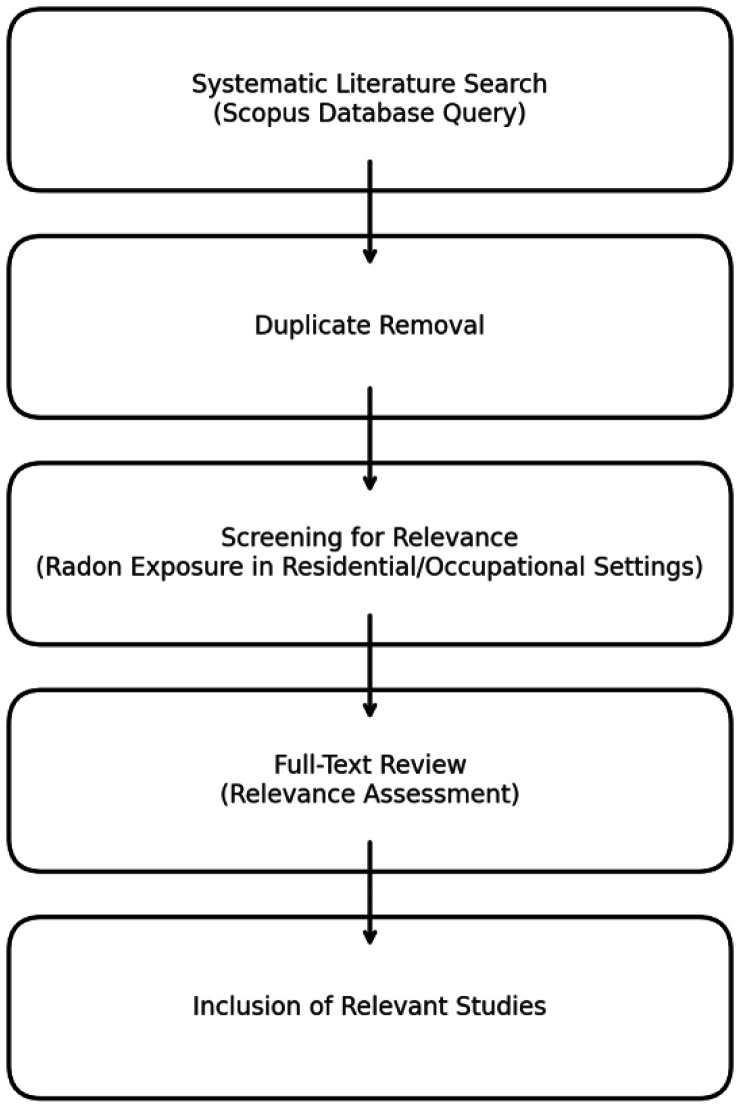
Flowchart of the systematic data collection and analysis process.

**Figure 2.**
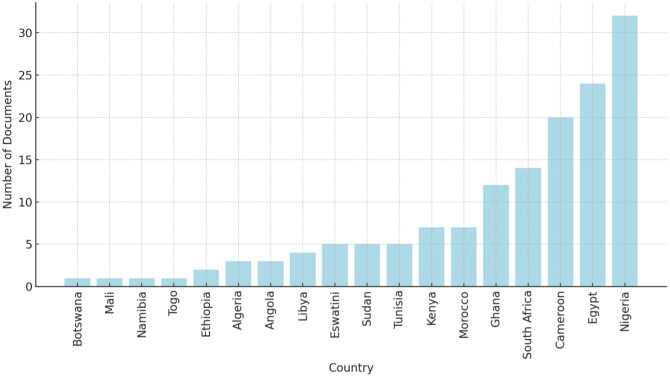
Number of documents per African country related to indoor radon research.

The query results are presented in Table 1 and Figure 2. The inclusion criteria for the articles were that they must be written in English and discuss indoor radon in African countries.

The remaining African countries, namely Benin, Burkina Faso, Burundi, Cape Verde, Central African Republic, Chad, Comoros, Democratic Republic of the Congo, Republic of the Congo, Cote d'Ivoire, Djibouti, Equatorial Guinea, Eritrea, Gabon, Gambia, Guinea, Guinea-Bissau, Lesotho, Liberia, Madagascar, Malawi, Mauritania, Mauritius, Mozambique, Niger, Rwanda, São Tome and Principe, Senegal, Seychelles, Sierra Leone, Somalia, South Sudan, Tanzania, Uganda, Zambia, and Zimbabwe, yielded no results for the keywords used in the search.

After the initial search, duplicate articles were removed, and the remaining articles were screened based on their title and abstract to determine their relevance. Then, full-text articles were obtained for the relevant studies, and further screening was conducted to ensure they met the inclusion criteria. The final set of articles was analyzed to identify the main themes and research gaps regarding indoor radon exposure in African countries.

**Table 1.** Results obtained with query from the Scopus database.

**Country**	**Nr. of documents**	**Time interval**	**References**
Botswana	1	2010	[22]
Mali	1	2013	[23]
Namibia	1	2018	[24]
Togo	1	2022	[25]
Ethiopia	2	2008–2021	[26]
Algeria	3	1990–2014	[27]
Angola	3	2020–2023	[28]
Libya	4	2010–2014	[29]
Eswatini (formerly Swaziland)	5	1994–1997	[30]
Sudan	5	2014–2021	[31]
Tunisia	5		[32]
Kenya	7	1999–2019	[33]
Morocco	6	2005–2022	[34]
Ghana	12	1992–2023	[35]
South Africa	14	2008–2023	[36]
Cameroon	20	2008–2023	[37],[38]
Egypt	24	1991–2022	[39]
Nigeria	32	2004–2022	[40]

## 3. General overview of the Geology of Africa

### 3.1. Framework

The study and knowledge of geology are essential to understand how radon gas emerges and concentrates, especially in a continent such as Africa, which presents a vast diversity of structural, lithological, and geogenic geology. Radon (specifically isotopes 220 and 222) is a naturally occurring radioactive gas that is part of the decay chains of uranium and thorium. As an inert, odorless, and colorless gas, radon can accumulate indoors, particularly in confined spaces such as homes and workplaces. Africa's geological formations significantly vary in their potential to generate radon due to radiogenic elements such as uranium and thorium found in certain rocks. For example, regions with significant volcanic activity, such as the East African Rift, or the uranium-rich deposits in Namibia, tend to have higher radon emissions. In Africa, there is a diverse range of geological formations and processes, including volcanic activity, sedimentary basins, and tectonic movements, which can affect the occurrence and concentration of radon gas. Therefore, understanding the geology of a particular region is crucial in predicting the potential for radon exposure and developing appropriate mitigation strategies. The geology of Africa is complex, and it is imperative to research and identify the areas with a higher radon potential to implement appropriate measures to protect public health. With the increasing awareness of the health risks associated with radon exposure, further research on the geology of Africa and its relation to radon occurrence is crucial to develop effective policies and strategies to reduce exposure and to mitigate the associated risks.

### 3.2. African geology remarks

The African continent is characterized by its geological diversity, with a wide variety of rock formations, geological features, and tectonic activity. The continent's rich mineral resources have played an important role in its economic development, while its geological features have shaped its landscapes and ecosystems. The continent is divided into several major geological regions, each one with its unique characteristics. Understanding the geology of this region is essential to identify potential radon sources and to develop strategies for radon mitigation and exposure reduction. Africa's diverse geology, including uranium-rich formations, volcanic regions, and sedimentary basins, is closely linked to radon risks. Regions such as the East African Rift and uranium mining areas in Namibia and South Africa should be prioritized for radon research and mitigation efforts. Mapping geological features alongside radon measurements will help identify high-risk areas and inform public health strategies. In summary, the geology of the African continent can be described for each of its constituent regions as follows [41]:

• The northern region of Africa is a vast and diverse area that spans several countries. In addition to the Atlas Mountains, several other geological features and formations are present in this region. In Morocco, the Atlas Mountains consist of several distinct geological units, including the Pre-Cambrian basement rocks, the Paleozoic and Mesozoic sedimentary rocks, and the Cenozoic volcanic and sedimentary rocks. These geological formations are rich in mineral resources, including lead, zinc, copper, and manganese, and some of these minerals can contribute to elevated levels of radon [42]. Algeria is home to several mountain ranges, including the Tell Atlas, the Saharan Atlas, and the Hoggar Mountains. These mountains are primarily composed of sedimentary rocks, with some volcanic and metamorphic rocks present in the Hoggar Mountains. In addition to the mountains, Algeria has several important geological features, including the Tassili n'Ajjer plateau, which is home to several ancient rock art sites. Tunisia also has several mountain ranges, including the Dorsal Atlas and the Saharan Atlas. The country is primarily composed of sedimentary rocks, including limestone, sandstone, and shale. In addition to the mountains, Tunisia has several important geological features, including the Chott el Djerid salt pan, which is one of the largest salt pans in the world. Libya is primarily composed of sedimentary rocks, including sandstone, shale, and limestone. The country is home to several important geological features, including the Acacus Mountains, which are known for their unique rock formations and ancient rock art. Egypt is home to several important geological features, including the Nile River and the Sinai Peninsula. The country is primarily composed of sedimentary rocks, including limestone, sandstone, and shale. In addition to the sedimentary rocks, there are also several important volcanic and metamorphic rocks present in the Sinai Peninsula.

• The Central region of Africa is vast and diverse, encompassing several countries with varying geologic characteristics. One of the most prominent features of the region is the Congo Basin, which spans several countries, including the Democratic Republic of the Congo, Central African Republic, the Republic of the Congo, Cameroon, and Gabon. The Congo Basin is a large sedimentary basin that contains the Congo River and some of the world's largest rainforests. It is underlain by Precambrian rocks, which are some of the oldest on the planet. The Democratic Republic of the Congo is one of the largest countries in the region and is known for its extensive mineral wealth. The country's geology is dominated by the Congo Basin, which is home to large deposits of copper, cobalt, and other minerals. The Katanga Supergroup, which is a sequence of sedimentary rocks that formed over 500 million years ago, hosts most of the country's copper and cobalt deposits. The Central African Republic is also situated in the Congo Basin and has significant mineral deposits, including gold, diamonds, and uranium. The country is underlain by Precambrian rocks, which are part of the West African Craton. The craton extends across several countries in the region, including Cameroon and Chad, and is one of the oldest and most stable pieces of continental crust in the world. The Republic of the Congo is situated to the west of the Democratic Republic of the Congo and is also underlain by Precambrian rocks. The country is known for its oil reserves, which are mainly found in sedimentary rocks that were deposited during the Cretaceous period. Cameroon is another country in the region that is situated on the West African Craton. The country's geology is dominated by volcanic rocks, which are associated with the Cameroon Volcanic Line. The volcanic line stretches from the Gulf of Guinea to Lake Chad and is home to several active and inactive volcanoes. Gabon is situated to the west of the Congo Basin and is known for its large oil reserves. The country's geology is dominated by sedimentary rocks, which were deposited during the Cretaceous period. Additionally, Gabon is home to some mineral deposits, including manganese and uranium. The Central region of Africa is characterized by its diverse geology and mineral wealth. The Congo Basin, which is one of the largest sedimentary basins in the world, is a dominant feature of the region and is home to significant mineral deposits. Moreover, the region is underlain by Precambrian rocks, which are some of the oldest on the planet, and host many important mineral deposits.

• The eastern region of Africa is a diverse area that is dominated by the East African Rift System, which is an extensive geological feature that stretches for thousands of kilometers from the Red Sea to Zimbabwe. The rift system is formed by the separation of the African Plate from the Arabian Plate, which has caused the stretching and thinning of the Earth's crust in this region. This geological activity has resulted in the formation of a series of rift valleys, volcanoes, and large lakes. Ethiopia, which is located in the eastern region of Africa, is home to a large part of the East African Rift System. It has a complex geological history and is characterized by numerous volcanic features, including the Danakil Depression, which is one of the lowest and hottest points on the planet. The country is also home to a series of large lakes, including Lake Tana and Lake Awasa. Kenya, which is located in the eastern region of Africa, is famous for its wildlife and is home to some of Africa's tallest mountains, including Mount Kenya, which is the second-highest peak on the continent. The country is also home to the Great Rift Valley, which is a series of interconnected rifts and valleys that are home to a variety of wildlife and volcanic features. Tanzania, which is also located in the eastern region of Africa, is home to Mount Kilimanjaro, which is the highest mountain on the continent. The country is also home to the Serengeti National Park, which is one of the most famous wildlife reserves in the world. Tanzania is also known for its large lakes, including Lake Victoria, which is the largest lake in Africa and the second-largest freshwater lake in the world. Uganda, which is located in the eastern region of Africa, is home to a portion of the East African Rift System and is characterized by a series of rift valleys and lakes, including Lake Victoria. Additionally, the country is home to several national parks and reserves, including Bwindi Impenetrable National Park, which is home to half of the world's population of endangered mountain gorillas. Mozambique, which is located in the southeastern part of Africa, is also part of the East African Rift System. The country has a long coastline along the Indian Ocean and is home to many large rivers, including the Zambezi River. Additionally, Mozambique is known for its mineral deposits, including coal, titanium, and natural gas. The East African Rift System has played a significant role in shaping the region's geology and has contributed to the formation of some of the world's most iconic landscapes, such as the Serengeti and the Great Rift Valley.

• The southern region of Africa is also known for its unique geology and diverse mineral resources. South Africa, in particular, is home to some of the world's largest reserves of gold, diamonds, and platinum group metals. The Witwatersrand Basin, located in South Africa, is the world's largest gold deposit and has been mined for over a century. Namibia is also known for its rich mineral resources, including diamonds, uranium, and copper. The country's geology is dominated by the Namib Desert, which is one of the driest places on earth and is home to a variety of unique flora and fauna. On the other hand, Botswana is dominated by the Kalahari Desert, which is home to a diverse range of wildlife, including the iconic African elephant. Additionally, the country is known for its diamond reserves, which are among the largest in the world. Zimbabwe is another country in the southern region of Africa that is rich in mineral resources, including gold, platinum, and diamonds. The country's geology is characterized by the Great Dyke, which is a layered mafic and ultramafic intrusion that is rich in minerals such as chromite, platinum, and nickel.

## 4. Literature review by country

### 4.1. Botswana

The article, “Indoor and outdoor radon levels and its diurnal variations in Botswana” by Murty, King, Karunakara, and Raju (2010), investigated both indoor and outdoor radon levels in Botswana, with a particular focus on the diurnal variations of radon concentrations [22]. Published in Nuclear Instruments and Methods in Physics Research Section A: Accelerators, Spectrometers, Detectors and Associated Equipment, this study is an essential reference to understand radon exposure patterns in the region. The authors of the study conducted measurements of indoor and outdoor radon concentrations, thereby assessing the differences between the two settings and analyzing the diurnal variations in radon levels. The study in Botswana utilized passive radon detectors, which were placed in indoor environments to monitor radon concentrations over a set period, thus allowing for an assessment of diurnal variations. The findings confirm that the indoor radon concentrations were higher than the outdoor concentrations, thus emphasizing the importance of monitoring and mitigating radon exposure in residential and commercial buildings.

### 4.2. Mali

In Mali, passive radon detectors were used to measure the indoor radon concentrations in selected dwellings, providing an estimate of long-term exposure. Traore et al. (2013) conducted an assessment of the activity and an effective dose rate of 222Rn in several dwellings in the city [23]. The study found that the average radon concentration in the dwellings was 42.3 Bq/m³, which was below the recommended action level of 200 Bq/m³. However, some dwellings had higher radon concentrations, with the maximum concentration reaching 218 Bq/m³. Additionally, the authors estimated the effective dose rate from radon exposure, which was found to be 1.1 mSv/year, and was below the recommended limit of 1 mSv/year for the general public. The authors noted that the factors that affected radon levels in dwellings in Bamako were likely to be similar to those in other cities in West Africa. They suggested that more studies were needed to better understand the distribution and sources of radon in the region and to develop effective strategies to mitigate the risks associated with radon exposure.

### 4.3. Namibia

In Namibia, a study by Munyaradzi et al. (2018) investigated the excess lifetime cancer risk due to natural radioactivity in soils in the Karibib town [24]. The study found that the natural radioactivity levels in soil samples collected from the town were generally higher than the world average. The authors estimated the excess lifetime cancer risk due to exposure to natural radioactivity in soil, which was found to be 1.9 × 10^−3^ for the general population and 6.7 × 10^−3^ for miners. The study suggested that measures should be taken to mitigate the risks associated with a long-term exposure to natural radioactivity in soil, especially for those working in the mining industry. Additionally, the authors suggested that further studies were needed to better understand the distribution and sources of natural radioactivity in soil in the region.

### 4.4. Togo

The article “Radiological assessment and statistical approaches of natural radionuclides in soil samples related to phosphate ore activities in the site of Dagbati, the southern region of Togo” by Hazou et al. (2022) examined the radiological impact of phosphate ore activities in the southern region of Togo [25]. The study assessed the concentrations of natural radionuclides in soil samples collected from the Dagbati site, where phosphate mining activities have been performed. The authors utilized various analytical techniques to measure the concentrations of radionuclides such as uranium-238, thorium-232, and potassium-40. Additionally, they employed statistical approaches to analyze the data obtained and assess the potential health risks associated with the presence of these radionuclides. The study found that the concentrations of natural radionuclides in the soil samples were within the range of values reported in other studies in different parts of the world. However, the concentrations of uranium and thorium were found to be higher in the samples collected from the mining area compared to those collected from the control area. The authors suggest that the higher concentrations of uranium and thorium in the soil samples from the mining area may be due to the mining activities. Moreover, the authors calculated the radiation hazard indices and effective doses associated with the presence of natural radionuclides in the soil samples. The results of the hazard indices were within acceptable levels, thus indicating no immediate health risk to the public. However, the effective dose calculations revealed that the population in the mining area may be exposed to slightly higher levels of radiation than those in the control area.

### 4.5. Ethiopia

Studies in Ethiopia have employed a range of techniques, including passive radon detectors and soil sample analyses, to assess the distribution of radon-222 and other natural radioactive elements in the region. In 2008, Tiruneh and Kebede conducted the first significant study on natural radioactivity measurements in the southern part of Ethiopia [26]. Their research presented preliminary results on the levels of radon and other natural radioactive elements in the region. By assessing the concentrations of radon-222, uranium-238, and thorium-232 in soil samples, the study provided important insights into the distribution of these radioactive elements and their potential impact on human health and the environment. This pioneering research laid the groundwork for future studies on radioactivity in Ethiopia. More recently, Degu Belete and Alemu Anteneh (2021) published a general overview of radon studies in health hazard perspectives [43]. Their paper reviewed the current state of radon research, focusing on the health risks associated with radon exposure. By synthesizing the findings of numerous studies, the authors provided a comprehensive understanding of the potential health hazards posed by radon exposure, including lung cancer risk.

### 4.6. Algeria

In Algeria, indoor radon measurements were conducted using solid-state nuclear track detectors (SSNTDs), thus allowing for long-term monitoring in selected residential and workplace environments. The initial study, conducted by Djeffal, Cherouati, and Djouider (1990), constituted a critical foray into the understanding of indoor radon exposure in the Algerian context [27]. Measurements of radon levels in selected regions were reported in Radiation Protection Dosimetry, thus highlighting the necessity of monitoring indoor radon concentrations to safeguard public health. This foundational study laid the groundwork for subsequent research endeavors in the domain. After the aforementioned study, Amrani and Cherouati (1999) shifted the focus to the health effects that resulted from radon-222 in drinking water in Algiers [44]. This investigation, which was featured in the Journal of Radiological Protection, probed the potential health hazards linked to radon exposure via drinking water. The findings accentuated the importance of monitoring radon levels in water sources, as well as understanding the subsequent ramifications for public health. In a more recent development, Aït Ziane, Lounis-Mokrani, and Allab (2014) broadened the scope of radon research in Algeria by assessing exposure to indoor radon and natural gamma radiation in select workplaces within Algiers [45]. Published in Radiation Protection Dosimetry, this study aimed to ascertain the exposure levels to radon and gamma radiation in diverse occupational settings.

### 4.7. Angola

In Angola, the studies primarily utilized SSNTDs to measure radon concentrations in indoor environments, as well as the radon potential in water sources. The historical perspective and content of three key articles provide valuable insights into radon and thoron concentrations in Angola, as well as their implications for public health and risk mapping. In a 2020 study, Kessongo et al. investigated the radon concentration potential in the water of Bibala municipality, Angola, and its consequences for public consumption [28]. The study revealed that the radon concentration levels in the water samples were below the WHO recommended guideline value of 100 Bq/L. However, the authors suggested that continued monitoring is necessary to ensure that the levels do not exceed the recommended values, which could pose a health risk to the public. Another study conducted by Bahu et al. in 2021 focused on the exposure to radon in buildings in the municipality of Lubango, Angola, during the winter months [46]. The study revealed that the indoor radon concentration levels were below the WHO recommended values of 300 Bq/m³. However, the authors suggested that continued monitoring is necessary to determine the annual average radon concentration levels and to assess the long-term health implications. In a more recent study published in 2023, Baptista et al. investigated the radon and thoron concentrations in the southwest region of Angola and their implications for risk mapping [47]. The study revealed that the radon concentration levels in the region were generally low, except for some specific areas where higher values were recorded.

### 4.8. Libya

One of the earliest works in this field was performed by Al Bosta et al. (2010), who investigated the indoor radon concentrations and the corresponding lung cancer risk in the Celein region, west of Al Khums, Libya [29]. The study found that the average indoor radon concentration was below the recommended action level of 200 Bq/m³, though there was still a potential risk of lung cancer due to long-term radon exposure. Following this initial assessment, Al-Azmi et al. (2012) conducted coordinated indoor radon surveys in some Arab countries, including Libya [48]. The study found that the indoor radon concentrations in the surveyed buildings were generally low, though some buildings had elevated levels of radon. The authors suggested that measures should be taken to mitigate the risks associated with a long-term exposure to radon in these buildings. Saad et al. (2013) investigated radon exhalation from Libyan soil samples measured with the SSNTD technique [49]. The study found that the radon exhalation rates from the soil samples were generally low, though there were some locations with elevated rates of radon exhalation. The authors suggested that further studies are needed to better understand the distribution and sources of radon in Libya. In a more recent study, Saad et al. (2014) assessed radon exhalation from building materials used in Libya [50]. The study found that the radon exhalation rates from the building materials were generally low, though some materials had higher rates of radon exhalation.

### 4.9. Eswatini (formerly Swaziland)

The first notable study on radon exposure in Swaziland was conducted by Nsibande, Mahlobo, and Farid (1994), who measured the radon levels inside residences in the country [30]. Their research provided valuable insights into the potential risks associated with indoor radon exposure in Swazi homes. Mahlobo, Nsibande, and Farid (1995) continued their investigation of indoor radon exposure by measuring the indoor 222Rn concentrations using SSNTD [51]. This study contributed to the development and application of SSNTD as a reliable to measure radon concentrations in the indoor environment. Farid (1995) further explored the use of passive track detectors for radon determination in indoor settings, thus highlighting the advantages of this method, which include an ease of use and a low cost [52]. This research helped establish passive track detectors as a widely accepted technique to assess indoor radon exposure. In 1996, Farid shifted the focus of radon research in Swaziland to the assessment of radon concentrations in groundwater [53]. By employing CR-39 nuclear track detectors, Farid's study revealed valuable information about radon levels in the country's water sources, thus providing important data to understand the potential health risks associated with radon exposure through water consumption. Farid (1997) conducted a comprehensive study on indoor and soil radon measurements in Swaziland using track detectors, thus effectively combining the research on residential, indoor, and groundwater radon exposure [54].

### 4.10. Sudan

In Sudan, several studies have been conducted to assess the radon levels and their associated risks. One such study was conducted by Elzain in 2014, where he studied the indoor radon levels and the radon effective dose in dwellings of some cities of Gezira State in Sudan [31]. The study found that the indoor radon levels in some dwellings exceeded the recommended limits set by the WHO, indicating a potential health risk for the occupants. In 2015, Idriss et al. characterized the 222Rn and meteorological parameters in Uro houses at the South Kordofan state [55]. The study found that the average indoor radon concentration was higher than the recommended limit, and the levels varied depending on the season and location of the houses. Elzain continued his research in 2015 and studied the radon exhalation rates from some building materials used in Sudan [56]. The study found that some of the materials had high radon exhalation rates, thus indicating a potential source of indoor radon. In 2017, Elzain determined the soil gas radon concentration from some locations of Gedarif town, Sudan, using CR-39 [57]. The study found that the soil gas radon concentration significantly varied depending on the location and depth of the soil. In 2021, Mbembe et al. assessed the environmental health risks due to the indoor radon levels inside workplaces in Sudan [58].

### 4.11. Tunisia

The history of radon studies in Tunisia dates back to the year 2000 when Mahjoubi et al. conducted a study titled “Estimation of the exposure related to the presence of radon-222 indoor phosphate treatment factories and their environments in Tunisia” [32]. This study was published in the journal Radioprotection and aimed to assess the exposure of workers to radon in phosphate treatment factories and their surrounding environments in Tunisia. In 2004, Michèle et al. conducted a study titled “Radon concentrations in some dwellings of Tunisia”, which was published in Health Physics [59]. The study aimed to measure the indoor radon concentrations in selected dwellings in Tunisia and to evaluate the associated health risks. In 2006, El May et al. conducted a study titled “Le radon dans les maisons tunisiennes”, which was published in Tunisie médicale [60]. This study aimed to measure the radon concentrations in Tunisian homes and assess the associated health risks. In 2010, Labidi et al. conducted a study titled “Radon in elementary schools in Tunisia”, which was published in Radioprotection [61]. The study aimed to measure the radon concentrations in elementary schools in Tunisia and assess the associated health risks. In 2012, Labidi et al. conducted a study titled “Indoor radon in Tunisian spas”, which was also published in Radioprotection [62].

### 4.12. Kenya

Over the past few decades, several studies have been conducted to assess human exposure to natural sources of radiation in Kenya. One of the earliest works in this field was carried out by Mustapha et al. (1999), who conducted an assessment of human exposure to natural sources of radiation in Kenya [33]. The authors found that the average annual dose of 1.4 mSv is above the recommended public exposure limit of 1 mSv/year, thus indicating a potential health risk that warrants mitigation measures. Following this initial assessment, Mustapha et al. (2002) conducted a preliminary report on radon concentration in drinking water and indoor air in Kenya [63]. The study found that the radon concentration in both the drinking water and indoor air was generally low, although some locations had elevated levels of radon. The authors recommended further studies to better understand the distribution and sources of radon in the country. Chege et al. (2009) investigated the influence of meteorological parameters on indoor radon in selected traditional Kenyan dwellings [64]. The study found that there was a significant correlation between the indoor radon concentrations and meteorological parameters, such as temperature and rainfall. The authors suggested that these factors should be considered when designing radon mitigation strategies in traditional Kenyan dwellings. In a more recent study, Chege et al. (2015) estimated the annual effective dose due to radon and thoron concentrations in mud dwellings of Mrima Hill, Kenya [65]. The study found that the estimated annual effective dose from radon and thoron exposure was below the recommended dose limit of 1 mSv per year for the general public. However, the authors suggested that further studies were needed to better understand the health effects of a long-term exposure to radon and thoron. In 2019, Nyambura et al. (2019) conducted an assessment of the annual effective dose due to radon and thoron progenies in dwellings of Kilimambogo, Kenya [66]. The study found that the estimated annual effective dose from radon and thoron exposure was generally low, although some households had elevated levels of radon and thoron progenies.

### 4.13. Morocco

One of the earliest works in this field was carried out by Oufni et al. (2005), who determined the radon levels and effective dose rates in Moroccan dwellings using SSNTDs [34]. The study found that the average radon concentration in the dwellings was 43.2 Bq/m³, which was below the recommended action level of 200 Bq/m³. The authors suggested that more studies were needed to better understand the distribution and sources of radon in the country. Following this initial assessment, Misdaq et al. (2012) investigated the radiation doses to individuals due to 238U, 232Th, and 222Rn from the immersion in thermal waters and to radon progeny from the inhalation of air inside thermal stations [67]. The study found that the effective dose rates from radon exposure were generally low, but some individuals had elevated doses due to their occupation or prolonged radon exposure. In a more recent study, Abdo et al. (2021) assessed radon exposure and its decay product aerosols in some houses in the province of El Jadida, Morocco [68]. The study found that the radon levels in the houses widely varied, and that the annual effective dose from radon exposure was generally low, although some houses had elevated doses due to their location or construction materials. Misdaq et al. (2019) measured radon, thoron, and their daughters in the air of marble factories and estimated the resulting alpha-radiation doses to the lungs of workers [69]. The study found that the workers in the marble factories had an increased risk of lung cancer due to their exposure to radon and its progeny. In more recent studies, Abdo et al. (2021) and Ouakkas et al. (2022) developed models to estimate the short-term and annual effective doses from radon exposure in primary schools and indoor environments in the Doukkala-Abda region of Morocco [70],[71].

### 4.14. Ghana

In 1992, Andam initiated research on indoor and soil radon measurements in a tropical climate [35]. His pioneering work assessed the radon levels in Ghana, thus laying the foundation for future studies in the region. Further research by Akortia et al. (2010) focused on the indoor radon gas levels in selected homes in the Greater Accra region [72]. Their findings contributed to the understanding of radon distribution in Ghana and highlighted the need for more comprehensive radon monitoring. Otoo et al. (2018) conducted a seasonal study of the indoor radon levels in buildings in the Accra Metropolis, thus providing valuable data on the variation of radon concentrations throughout the year [73]. In a related study, they investigated the radon exhalation rates and natural radioactivity in construction materials used within the Greater Accra Region, thus contributing to the assessment of public exposure to radon [74]. Kitson-Mills et al. (2019) assessed the indoor radon levels in a suburb of Ghana, thus further expanding the knowledge of radon distribution in the country [75]. Nsiah-Akoto et al. (2019) detailed the Ghanaian strategy for indoor radon mapping, which has been instrumental in understanding radon exposure and developing mitigation strategies [76]. Opoku-Ntim et al. (2020) examined the indoor radon concentrations and risk assessment in dwellings in Obuasi, a mining town, while Otoo et al. (2020) studied the radon levels, radium concentration, and estimated effective doses in dwellings and soils in gold mining towns in the Eastern Region of Ghana [77],[78]. Kpordzro et al. (2022) assessed the seasonal radon concentration in dwellings and soils in selected areas in Ga East, Greater Accra Region, while Loffredo et al. (2022) applied the Gini method to an indoor radon survey in Kpong, Ghana [79],[80]. Otoo et al. (2022) conducted a correlation analysis of natural radionuclides, radon exposure, soil particles, and moisture from quarry towns in the Greater Accra Region [81]. Most recently, Otoo et al. (2023) investigated radon mapping, radium correlation, seasonal indoor radon, and radon exhalation levels in communities around the Ghana Atomic Energy Commission [82].

### 4.15. South Africa

In 2008, Lindsay, Newman, and Speelman conducted a study in Paarl, South Africa, using electret ion chambers and gamma-ray spectrometry to measure the airborne radon levels in houses and the associated source terms [36]. A year later, Kgabi, Mokgethi, and Bubu investigated the atmospheric radioactivity associated with gold mining activities in the North West Province of South Africa [83]. In 2016 and 2017, Botha, Newman, Maleka, and Lindsay conducted several studies in Montagu and selected wine cellars in the Western Cape, thereby measuring the radon and thoron levels in the air and the associated effective doses for occupational exposure [84],[85]. In 2017, Winde, Erasmus, and Geipel revisited a case study of uranium-contaminated drinking water linked to leukemia, thereby taking alternative exposure pathways into account [86]. Bezuidenhout conducted two studies in 2019 and 2021, and estimated the radon potential through the measurement of uranium concentrations in granite geology and geological units in South Africa [87],[88]. Additionally, in 2019, Moshupya, Abiye, Mouri, Levin, Strauss, and Strydom assessed the radon concentration and its impact on human health in a region dominated by abandoned gold mine tailings dams [89]. In 2020 and 2021, le Roux, Bezuidenhout, Smit, and Newman studied the indoor radon measurements in the South African West Coast peninsula and Secunda, Mpumalanga Province, respectively [90],[91]. Strydom, Nel, Petersen, Nel, and Ramjukadh used radon isotopes to detect groundwater discharge in streams draining Table Mountain Group (TMG) aquifers [92]. In 2022, le Roux investigated the effect of the coal industry on the indoor radon concentrations in eMalahleni, Mpumalanga Province, while Lindsay, Mngonyama, Molahlehi, Ngwadla, and Ramonnye conducted a pilot study of the thoron concentration in an underground thorium mine [93],[94]. Lastly, in 2023, Maheso, Bezuidenhout, and Newman measured the indoor radon levels in homes and schools in the Western Cape, South Africa, using a school science outreach initiative and corresponding model predictions [95].

### 4.16. Cameroon

The study of natural radiation exposure, including radon, has been a significant focus in Cameroon, as demonstrated by numerous articles published between 2008 and 2023. These studies cover various aspects of radon research, including indoor and outdoor measurements, correlations with different materials, and dose assessments for the public. In 2008, Ngachin et al. investigated the radon exhalation rate and radioactive content in construction materials [37]. This study highlighted the importance of understanding radon exposure from building materials, which is a theme further expanded upon in later studies. From 2014 to 2019, a series of articles focused on the indoor radon measurements in different regions of Cameroon, such as uranium-rich areas, oil-bearing regions, and mining locations [38], [96]–[102]. Researchers, including Tchuente et al., Abiama et al., and Mbembe et al., assessed the exposure to radon in these areas and its impact on public health [96],[97],[103]. Simultaneous measurements of radon and thoron, conducted by Serge Didier et al. [100], emphasized the importance of understanding both isotopes in indoor environments. In 2018 and beyond, researchers expanded their focus to include external dose estimation and natural radioactivity measurements in various regions. Bouba O et al. utilized car-borne surveys to assess radiation exposure in Douala city [104]. Later, Saïdou et al. explored indoor radon measurements using different detector types in bauxite-bearing areas [105]. More recently, studies have investigated radon exposure in specific contexts, such as water sources, soil gas radon, and building materials. These investigations, led by researchers such as Mbida Mbembe et al., Djeufack et al., Sadjo et al., Bachirou et al., Dieu Souffit et al., and Ndjana Nkoulou II et al., offer crucial insights into radon exposure risks and their mitigation [58],[106]–[115].

### 4.17. Egypt

In the early 1990s, Kenawy and Morsy (1991) conducted one of the first measurements of environmental radon concentrations in both indoor and outdoor settings in Egypt [39]. This was followed by Hassib et al. (1993), who assessed the radon concentrations in Egyptian dwellings using a passive technique [116]. During the late 1990s and early 2000s, research focused on the radon concentrations in specific environments, such as archaeological sites in Alexandria (Hafez et al., 1997), as well as indoor air (Mohammed, 1999) and Cairo, where a study on the domestic radon concentration and childhood cancer was conducted (Maged et al., 2000) [117]–[119]. Arafa et al. (2002) investigated the airborne radon concentrations in an Egyptian village, thus highlighting the importance of understanding radon exposure in rural settings [120]. In the mid-2000s, researchers began assessing the occupational exposure to radon, particularly among phosphate mine workers (Khater et al., 2004) [121]. El-Hussein (2005) studied natural radiation exposure in different realistic living rooms, while Maged and Ashraf (2005) examined the radon exhalation rates of building materials used in Egypt [122],[123]. Mohamed (2005) and Ghany (2006) continued investigating the radon levels in Egyptian dwellings, thus leading to a more comprehensive understanding of indoor radon exposure [124],[125]. From 2008 onwards, studies on radon exposure and the associated lung cancer risks were conducted, such as the research by El Gamal and Hosny (2008), which assessed the risk due to exposure from coastal sediments [126]. Moreover, seasonal variations of the indoor radon concentrations were explored (Abd El-Zaher, 2011) [127]. In 2012, Al-Azmi et al. coordinated indoor radon surveys in several Arab countries, including Egypt, to better understand the regional radon exposure [48]. Later studies focused on evaluating the radiological hazard indexes in building materials (Hassan et al., 2013) and the indoor radon concentration measurements at the site of the first nuclear power plant in Egypt (Hussein, 2014) [128],[129]. In 2015, Yuness et al. highlighted the importance of the indoor radon progeny activity in dose assessments [130]. Research on the radiological hazards of various materials continued, with Mostafa (2016) assessing the potential hazards caused by marble and granite tails, and Fares (2017) evaluating the environmental radioactivity impacts in Sinai [131],[132]. Wagdi et al. (2018) explored the indoor air quality indexes for preoccupancy assessments [133]. More recent studies have continued to investigate radon exposure in various settings, such as historical Roman buildings in southeastern Egypt (Hanfi et al., 2021) and radiation health hazard assessments among greenhouse farmers (Abd El-Zaher, 2022) [134],[135].

### 4.18. Nigeria

Over the past two decades, several studies have been conducted in Nigeria to assess the levels of indoor and outdoor radiation in various locations. Mokobia and Balogun (2004) measured the background gamma terrestrial dose rate in Nigerian functional coal mines [40]. Obed et al. (2010, 2011, 2012) conducted indoor radon surveys on university campuses, secondary schools, and dwellings in different regions of Nigeria [136]–[138]. Ajayi and Olubi (2016) investigated indoor radon levels in some dwellings in southwestern Nigeria [139]. Afolabi et al. (2015) studied the radon levels on a Nigerian university campus, while Ademola and Obed (2016) determined the soil radioactivity, indoor radon concentration levels, and their correlation in a specific region of southwestern Nigeria [140],[141]. Other studies have focused on specific locations and their potential radiation risks. For example, Okeji and Agwu (2012) assessed the indoor radon concentration in phosphate fertilizer warehouses in Nigeria, while Arabi et al. (2017) conducted a radiochemical evaluation of the groundwater around Mika uranium mineralization and environs [142],[143]. Faweya et al. (2019) evaluated the radon exhalation rates and the excessive lifetime cancer risk in dumpsites in Ondo City, southwestern Nigeria [144]. Adewoyin et al. (2019) investigated the indoor concentration of 222Rn and its possible health implications on the staff of a pharmaceutical company in Ogun State, Nigeria, and mapped the uranium-238 deposit and its contribution to indoor radon gas in Ota, Ogun State [145],[146]. Additionally, several studies have examined the relationship between the building characteristics and radon levels. For instance, Usikalu et al. (2017, 2018, 2020) monitored the radon concentration in different building types at Covenant University, Nigeria [147]–[149]. Aladeniyi et al. (2020) conducted a radiometric evaluation of the indoor radon levels, with an influence of building characteristics in residential homes from southwestern Nigeria [150]. Adegun et al. (2019) surveyed the indoor radon concentration in bank basements in three Nigerian cities [151]. Ajayi et al. (2019) surveyed the indoor radon levels in some universities in southwestern Nigeria, while Oni et al. (2022) conducted a preliminary probe of the radon content in drinking water in Ibadan, southwestern Nigeria [152],[153]. Finally, some studies have explored the use of different techniques to measure radiation levels. Olaoye et al. (2017, 2021) assessed the indoor radon levels in selected locations within Lagos State University, Ojo, Lagos, and estimated radon excess lung cancer near some dumpsites in Lagos, Nigeria [154],[155]. Khandaker et al. (2021) determined the radon concentration in the groundwater of Gadau, Bauchi State, Nigeria, and estimated the effective dose [156]. Ndubisi et al. (2021) analyzed the indoor radon level and its health risks parameters in three selected towns in Port Harcourt, Rivers State, Nigeria [157]. Asere et al. (2021) assessed excess gamma dose exposure levels in typical Nigerian commercial building materials distribution outlets [158]. Oladapo et al. (2022) evaluated the soil-gas radon concentrations from different geological units with varying strata in a crystalline basement complex of southwestern Nigeria, and Oni et al. (2022) investigated meteorological and geological influences on the indoor radon concentration in selected tertiary institutions in southwestern Nigeria using artificial neural network modelling [159],[160].

## 5. Discussion

In regions such as Europe, North America, Japan, and South Korea, comprehensive programs have been implemented to mitigate the health risks associated with radon exposure. These include public awareness campaigns, mandatory radon testing in homes and workplaces, and remediation programs where the radon levels exceed the recommended thresholds. For example, the EU's Radon Action Plan encourages member states to test buildings and reduce radon levels through construction techniques, while the United States Environmental Protection Agency (EPA) has set a 148 Bq/m³ action level for remediation. Similar programs in Japan and South Korea focus on setting guidelines for radon in homes and workplaces to protect public health.

The analysis of the articles found that there is a significant lack of research on radon exposure in most African countries. From the analysis presented, it is clear that a few countries, notably Cameroon, Egypt, and Nigeria, lead in terms of the number of radon-related publications. These countries have made significant contributions to the body of research, highlighting their awareness of and response to radon risks. However, the number of published articles remains small compared to other regions of the world. The primary focus of research on radon in African countries has been on monitoring and mitigating radon exposure in indoor and outdoor settings, with the ultimate goal of improving public health and reducing the associated risks. Another important theme is the radiological assessment and statistical analysis of natural radionuclides in the soil, water, and building structures, particularly concerning mining and other industrial activities. However, based on the available studies, the overall residential radon risk in Africa appears to be relatively low in many areas, particularly where homes are well-ventilated and located away from radon-emitting geological formations. However, the occupational radon risk is notably higher, especially in regions with extensive mining and underground activity. Workers in industries such as uranium mining may face a significantly higher exposure to radon, thus emphasizing the need for targeted radon monitoring and mitigation strategies in such workplaces.

Occupational radon exposure also features prominently in the literature, as various contexts, including phosphate treatment plants, mines, and workplaces, are examined to understand the extent of exposure and to implement protective measures. There is also a growing interest in exploring factors that affect the radon levels, such as the building materials, local geology, and industries, underscoring the necessity for additional research to develop effective mitigation strategies. Lastly, a crucial area of investigation concerns the human health impacts which result from radon exposure, including the lifetime cancer risk, which highlights the importance of devising mitigation strategies and ensuring ongoing monitoring to protect public health.

From the analysis of all the articles found for African countries where indoor radon research has been conducted, it becomes evident that several key themes emerge in the field, which are not so different from the subjects studied in other parts of the world. Among these, the primary focus is on monitoring and mitigating the indoor radon exposure, with the ultimate goal of improving public health and reducing the associated risks. Significant interest exists in exploring factors that affect the indoor radon levels, such as building materials, local geology, and industries, underscoring the necessity for additional research to develop effective mitigation strategies.

Despite the well-established health risks associated with radon exposure, there is a paucity of research on radon in many African countries. Benin, Burkina Faso, Burundi, Cape Verde, Central African Republic, Chad, Comoros, the Democratic Republic of the Congo, the Republic of the Congo, Cote d'Ivoire, Djibouti, Equatorial Guinea, Eritrea, Gabon, Gambia, Guinea, Guinea-Bissau, Lesotho, Liberia, Madagascar, Malawi, Mauritania, Mauritius, Mozambique, Niger, Rwanda, São Tome and Principe, Senegal, Seychelles, Sierra Leone, Somalia, South Sudan, Tanzania, Uganda, Zambia, and Zimbabwe are among the African countries where little or even no research has been conducted on radon. Other countries, including Botswana, Mali, Namibia, Togo, Ethiopia, Algeria, Angola, Libya, Eswatini (formerly Swaziland), Sudan, Tunisia, Kenya, Morocco, Ghana, South Africa, Cameroon, Egypt, and Nigeria, already present some research on this issue. While some studies have been conducted in these countries, the number of published articles on radon remains small, thus indicating that the field is still in its early stages.

The limited research on radon in these countries can be attributed to many factors. First, many African countries lack the resources and expertise necessary to conduct extensive research on environmental health issues such as radon exposure. Additionally, there may be a lack of awareness among policymakers and the general public about the health risks associated with radon exposure, which may contribute to a lack of funding for research. Moreover, the lack of standard protocols and guidelines to measure and assess radon exposure in these countries may be a contributing factor. This can make it difficult for researchers to compare results and for policymakers to develop effective regulations and policies to protect public health. This is further exacerbated by the lack of political stability and institutional capacity in many African countries, which makes it difficult to develop and implement policies to manage radon exposure. Countries with similar economic challenges, such as those in Southeast Asia, have successfully implemented low-cost radon testing and mitigation strategies. For instance, the use of passive radon detectors and public education campaigns have proven effective in reducing the radon levels in countries such as Bangladesh and India. African countries could benefit from adopting these cost-effective approaches to address the growing public health concern posed by radon exposure.

Another reason for the lack of research on radon in African countries is the relatively underexplored nature of the topic. Researchers have focused more on other environmental pollutants, and there is a general lack of awareness among policymakers and the general public regarding the health risks associated with radon exposure. The study of radon requires experts and trained personnel in the field of radiation protection and measurement. It is possible that there is a shortage of such experts and equipment to measure the radon levels in these countries. Many of these countries face significant economic challenges, and their resources are often directed toward more pressing concerns, such as poverty reduction, infrastructure development, and infectious disease control. Funding for research in radiation protection and measurement may not be a priority, and as a result, radon research may be considered a low priority. To address the gaps in radon research in Africa, several steps should be taken. First, there is a need for increased funding for environmental health research, particularly in under-resourced countries. Additionally, regional collaborations could facilitate the sharing of expertise and resources, such as mobile testing units and radon mitigation technologies. Moreover, governments should develop national radon action plans, modeled after the successful frameworks used in Europe and North America, to standardize testing and awareness programs.

The limited research on radon in many African countries is a cause for concern (Table 2). Given the well-established health risks associated with radon exposure, policymakers and researchers in these countries must prioritize the study of radon and develop the necessary frameworks and standards to manage its exposure. This will require increased awareness among policymakers and the public regarding the risks associated with radon, as well as increased investment in facilities and equipment for radon measurement and mitigation. Ultimately, this will lead to a healthier and safer living environment for people in these countries.

## 6. Conclusions

The analysis of indoor radon research in African countries revealed significant gaps in monitoring, mitigation, and understanding of radon exposure. Despite the established health risks, research in this area remains limited, with few studies conducted and published, thus indicating that the field is still underdeveloped. Contributing factors include a lack of resources, awareness, standard protocols, political stability, institutional capacity, expertise, equipment, and competing funding priorities. To address these challenges, it is essential to prioritize radon research and management in African countries. This includes developing frameworks and standards, increasing public and policymaker awareness, and investing in facilities and equipment for radon measurement and mitigation. Insights from regions such as Europe, North America, and parts of Asia, where robust radon management frameworks exist, can guide African countries to adopt cost-effective mitigation strategies and tailored policies. Key actions include integrating radon testing into public health policies, especially in high-risk geological areas, and conducting awareness campaigns to inform the public about radon exposure risks. Examples such as Tunisia's monitoring of radon in phosphate factories provide valuable models for industrial settings. International collaboration, particularly with organizations such as the International Atomic Energy Agency (IAEA), can support training and resource provision for local researchers, thus facilitating widespread radon testing and mitigation efforts. These initiatives are critical to create healthier living environments and to support sustainable development across the continent.

**Table 2.** Comparison of radon research and mitigation programs in African countries vs. Europe, North America, Japan, and South Korea.

**Region**	**Radon Action Plan**	**Public Awareness**	**Testing Mandates**	**Mitigation Strategies**	**Research Activity**
Africa (18 countries)	Very limited, only a few countries have initial plans (e.g., Tunisia, Sudan)	Low awareness, minimal campaigns	Few mandatory testing programs	Limited mitigation strategies available	Low research activity, mostly concentrated in a few countries
Europe	Comprehensive plans in most countries (EU directive 2013/59/Euratom)	High awareness with national campaigns	Mandatory testing in several countries	Extensive, supported by government incentives	High research activity with extensive academic and governmental support
North America	Comprehensive (EPA in the US, Canadian Radon Action Plan)	High awareness, EPA-driven campaigns	Mandatory in high-risk areas (US and Canada)	Established, with subsidies for remediation	High research activity with support from agencies like EPA
Japan	Established guidelines by the Ministry of Health, Labour, and Welfare	Moderate awareness	Mandatory testing in certain areas	Regulated, with focus on remediation in high-risk areas	Moderate research activity with focus on indoor radon levels
South Korea	National regulations and public health guidelines	Moderate awareness	Regulated testing in homes and workplaces	Similar to Japan, with an emphasis on regulated remediation	Moderate research activity focused on high-risk areas

## Use of AI tools declaration

The authors declare they have not used Artificial Intelligence (AI) tools in the creation of this article.
